# Exendin-4 as a Versatile Therapeutic Agent for the Amelioration of Diabetic Changes

**DOI:** 10.34172/apb.2022.025

**Published:** 2021-01-31

**Authors:** Hadi Rajabi, Mahdi Ahmadi, Somayeh Aslani, Shirin Saberianpour, Reza Rahbarghazi

**Affiliations:** ^1^Koc University Research Center for Translational Medicine (KUTTAM), Koc University School of Medicine, Istanbul, Turkey.; ^2^Tuberculosis and Lung Diseases Research Center, Tabriz University of Medical Sciences, Tabriz, Iran.; ^3^Department of Biochemistry and Clinical Laboratories, Faculty of Medicine, Tabriz University of Medical Sciences, Tabriz, Iran.; ^4^Vascular and Endovascular Surgery Research Center, Mashhad University of Medical Sciences, Mashhad, Iran.; ^5^Stem Cell Research Center, Tabriz University of Medical Sciences, Tabriz, Iran; ^6^Department of Applied Cell Sciences, Faculty of Advanced Medical Sciences, Tabriz University of Medical Sciences, Tabriz, Iran

**Keywords:** Diabetes complications, Exendin-4, Signaling pathways

## Abstract

Type 2 diabetes mellitus (T2DM) is a chronic metabolic abnormality leading to microvascular and macrovascular complications. Non-insulin Incretin mimic synthetic peptide exendin-4 was introduced as an anti-diabetic drug which helped diabetic patients with triggering insulin secretion; further researches have revealed an effective role of exendin-4 in treatment of T2DM related diseases. Exendin-4 is approximately similar to Glucagon-like peptide, thus it can bind to the glucagon-like peptide-1 receptor (GLP-1R) and activated different signaling pathways that are involved in various bioactivities such as apoptosis, insulin secretion and inactivation of microglial. In this review, we investigated the interesting role of exendin-4 in various kinds of T2DM related disorders through the activation of different signaling pathways.

## Introduction

**Figure 1:**
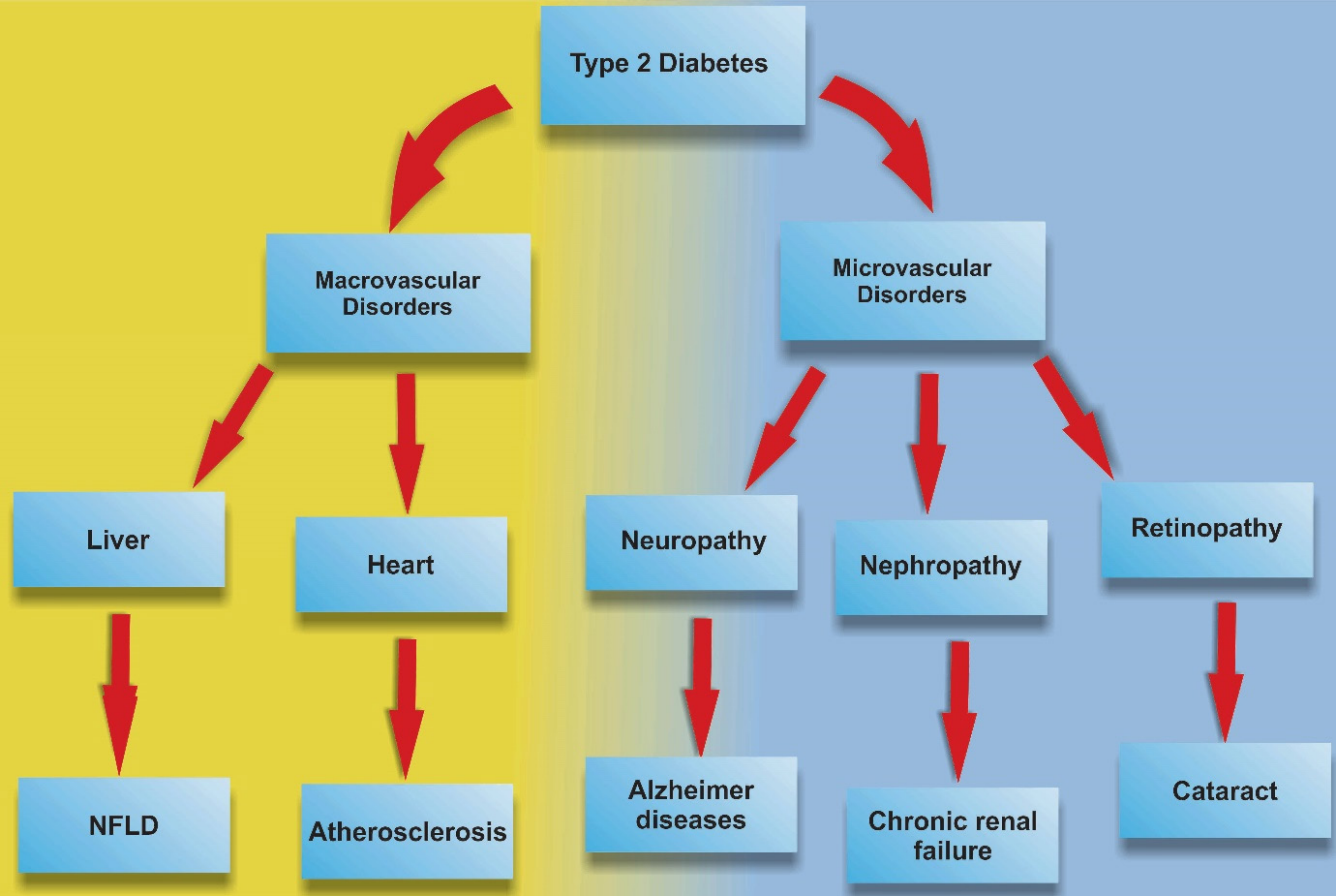
Type 2 Diabetes and related disorders.

**Figure 2:**
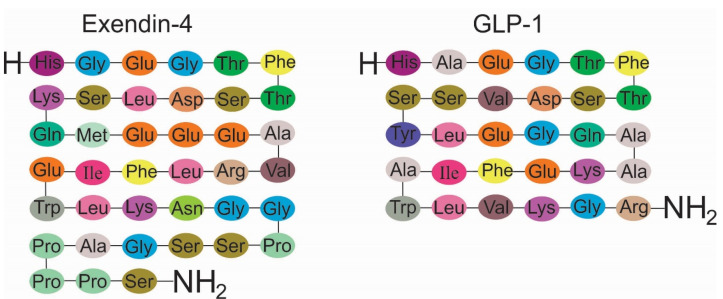
Exendin-4 shares 53% similarity with Glucagon-like peptide-1.

Type 2 diabetes mellitus (T2DM) is a progressive metabolic disorder with a high rate of prevalence worldwide contributing to profound socio-economic implications.^1^ T2DM is mainly identified by a hyperglycemic condition that is caused by the combination of cell resistance to insulin and/or insufficiency of pancreatic β cell to synthesize and release insulin to the systemic circulation in response to high levels of glucose. Irrespective of diabetic conditions, the imbalance of insulin could promote microvascular and macrovascular pathologies.^2^ The use of traditional approaches such as metformin administration and insulin therapy are the most common and prominent options for patients with T2DM (Table 1).^3,4^ However, numerous side effects have been reported in subjects with T2DM (Figure 1). In the case of pharmacological approaches, exendin-4 belongs to the incretin family is a glucagon-like peptide-1 receptor (GLP-1R) agonist with an ability to control hyperglycemic conditions in patients with T2DM and approved by food and drug administration and conceived as anti-diabetic agent.^5,6^ In the pharmaceutical industry, Exenatide is the synthetic form of exendin-4 with a 39 amino acid similar to GLP-1commonly seen in the saliva of the Gila monster (Figure 2).^7,8^

**Table 1:** Various medications which normally used for treating T2DM

Medications	Mechanism of action	Side effects
Metformin	Enhances insulin sensitivity in liver and peripheral tissues by activation of AMP activated protein kinase	Rare lactic acidosis Increases the risk of vitamin B-12 deficiency Gastrointestinal problems such as nausea and diarrhea
Sulfonylureas	Activates sulfonylurea receptor on beta cell to stimulate endogenous insulin secretion	Increased risk of cancer-related mortality compared with metformin
Insulin	activates insulin receptors to regulate metabolism of carbohydrate, fat and protein	Increased risk of cancer-related mortality compared with metformin Hypoglycemia risk
Thiazolidinedione Pioglitazone (Actos) Rosiglitazone (Avandia)	Enhances insulin sensitivity in peripheral tissues and liver by activation of peroxisome proliferator-activated receptor-gamma receptors	Cardiovascular effects Long-term using thiazolidinedione Increased risk of bone fractures in diabetic women not in men
Dipeptidyl peptidase 4 inhibitor	improves incretin pathway activation via inhibition of enzymatic breakdown of endogenous GLP-1 and gastric inhibitory peptide	Neurogenic inflammation increase in blood pressure, Enhanced inflammation and allergic reactions
Meglitinides	Increase insulin secretion during the early phase of insulin release	Weight gain (up to three kg in three months) Rare Diarrhea Frequent but not acute hypoglycemia

GLP-1 is formed by the post-translational stage of pro-glucagon hormone in the L-cells of the small intestine in response to the ingestion of food.^9^ GLP-1 has the potential to reduce systemic glucose levels by inducing insulin secretion. This factor also delays gastric emptying, inhibits glucagon secretion and reduces food intake rate.^10^ GLP-1 exerts its effects through GLP-1R.^8^ GLP-1R is a G protein-coupled receptor from glucagon receptor family by located on chromosome 6.^11,12^ Several studies have confirmed that GLP-1R is not exclusive for pancreatic tissue but also seen in the peripheral tissues such as lungs, stomach, intestines, kidneys, and heart in addition to the most areas of the brain.^13^ This receptor contains two major domains ECD (extracellular domain) and TM (transmembrane domain).^14^ As previously documented in different experiments, it demonstrated that EX-4 regulates glucose level in diabetic subjects via triggering insulin secretion, reducing glucagon secretion and food intake.^15-17^ Compared to GLP-1, EX-4 is resistant to dipeptidyl peptidase-IV activity, thereby possess a much longer plasma half-life and activity.^18^

In spite of the similarity between EX-4 and GLP-1 function and structure, there are some differences in the binding capacity to GLP-1R. The isolated GLP-1R ECD binds EX-4 with high affinity rather than GLP-1. Even, GLP-1 could attach with high affinity to TM part of GLP-1R with site-directed mutagenesis as compared with EX-4. In the case with GLP-1 N-terminal truncation, EX-4 loses affinity to attach this receptor but the interaction of GLP-1 with GLP-1 remains unaffected.^19^ However, there are some controversies related to EX-4 and GLP-1 interaction with GLP-1R. For instance, de Maturana et al showed the 400-fold binding affinity of Ex-4 (K_d_ = 6 nM) to the N-terminal domain of GLP-1R compared to GLP-1-GLP-1R attachment.^20^

## EX-4 related signaling pathway (s)

**Figure 3:**
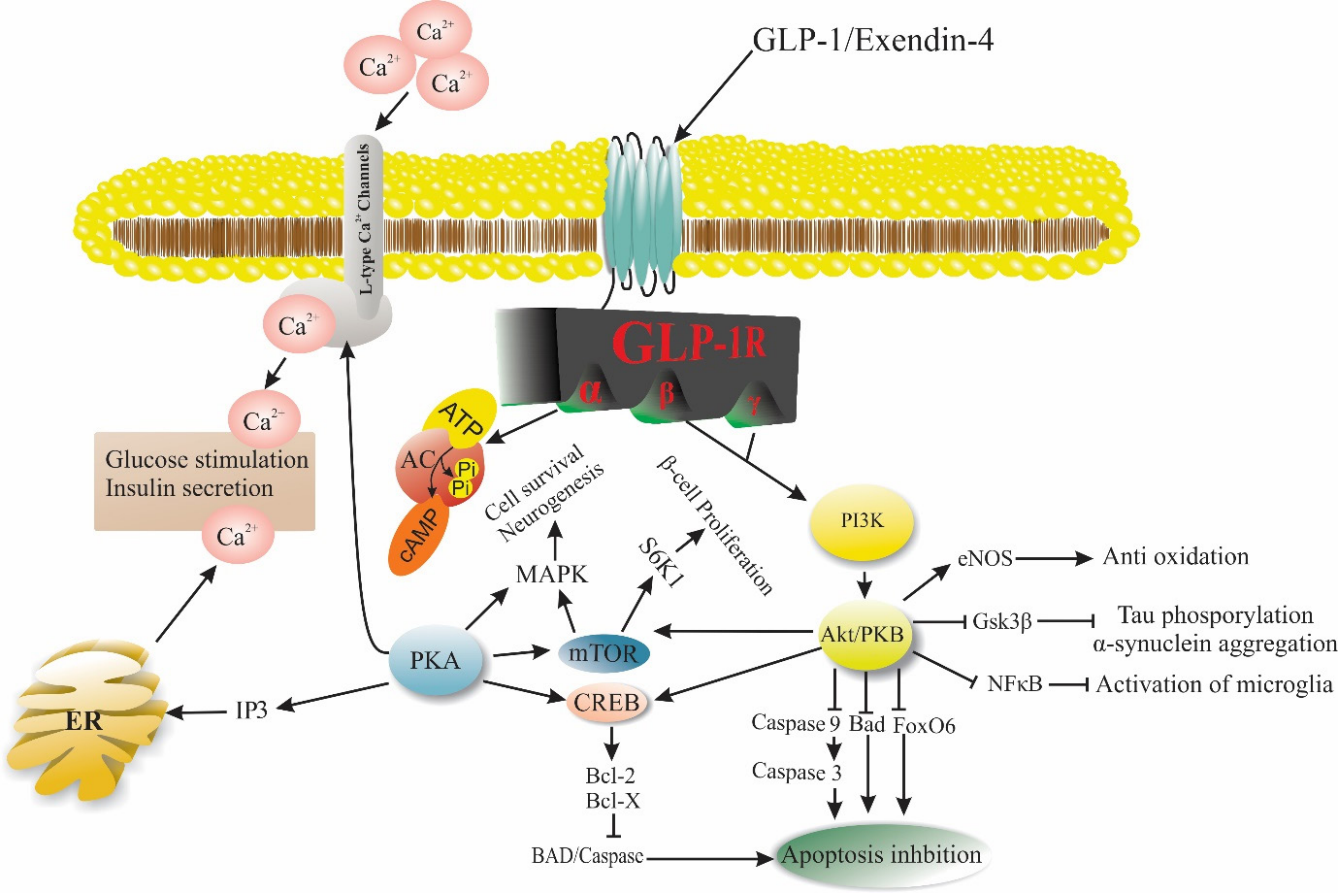
Exendin-4, a GLP-1R agonist plays a vibrant role in various biological activities such as apoptosis, Beta-cells proliferation, and glucose through different signaling pathways. Abbreviations: GLP-1: Glucagon-like peptide-1, GLP-1R: Glucagon-like peptide-1 receptor, AC: Adenylyl cyclase, PKA: Protein kinase A, IP3: Inositol 3 phosphate, ER: Endoplasmic reticulum, PI3K: Phosphoinositide 3-kinase, PKB/Akt: Protein kinase B, eNOS: Endothelial nitric oxide, Gsk3β: Glycogen synthase kinase 3 beta, NFκB: nuclear factor kappa-light-chain-enhancer of activated B cells, FoxO6: Forkhead Box O6, Bad: Bcl-2-associated death promoter, CREB: cAMP response element-binding, mTOR: mechanistic target of rapamycin, MAPK: mitogen-activated protein kinase, S6K1: Ribosomal protein S6 kinase beta-1.

The critical activity of EX-4 correlates with the induction of multiple downstream effectors from different signaling pathways.^21^ As previously mentioned, different biological processes such as cell proliferation and apoptosis modulation are initiated through activation of phosphatidylinositol 3-kinase/Akt (PI3K/AKT) adenylyl cyclase/protein kinase A signaling pathways.^22^ In PI3K/AKT axis, the activation of PI3K phosphorylates Akt factor which in turn affects the dynamic of different factors.^23^ For example, it was mentioned that activated Akt could increase the level of nitric oxide (NO) by phosphorylation of endothelial nitric oxide (eNOS) and contributes to the modulation of nitrosative stress.^24^ Of note, EX-4 has the potential to regulate the function of neurons by the suppression of glycogen synthase kinase-3β (GSK-3β). The decrease of GSK-3β function declines Tau protein phosphorylation thereby prohibits the aggregation of α-synuclein. Therefore, EX-4 is able to modulate the function of neurons and endothelial cells (ECs) during pathological conditions. It is noteworthy to mention that the application of EX-4 could decrease the cytopathic effects of Parkinson’s and Alzheimer’s diseases. In addition, it was well-established that the activation of microglial nuclear factor-ƙB (NF-ƙB) after administration of EX-4 provokes pro-inflammatory responses. One of the possible therapeutic effects of EX-4 on the injured cells could be related to the modulation of apoptosis. It was determined that the activation of Akt by EX-4 inhibits Caspase cascade such as caspase-9 and -3. In addition, the inhibition of pro-apoptotic factors such as Bad and Forkhead box O (FOXO) transcription factors decreased apoptotic changes.^25^ The above-mentioned factors are thought to participate in different cell activities (Figure 3). For instance, the critical role of EX-4 was determined in the regulation of β-cell proliferation via PI3K/Akt and mTORC1/S6K1-dependent signaling pathway.^26,27^ In contrast to the induction of cell proliferation, and increased proliferation rate could be correlated with the abortion of lipotoxicity-induced apoptosis after induction of protein kinase B and inhibition of mitochondrial-derived Caspase 3 while the transcription of Bcl-2 was found to up-regulate under these conditions.^28,29^ In addition to the therapeutic effects of EX-4 on insulin-producing cells, exposure of human endothelial cell lines to EX-4 pre-treated with apoptosis inducer tunicamycin decreased the possibility of atherosclerosis by reducing phosphorylated inositol requiring enzyme-1α/inositol requiring enzyme-1α ratio. Phosphorylation of c-JNK is also diminished in EX-4-treated human endothelial cell lines.^30^ Considering the detrimental effects of accumulated free acids in the promotion of endothelial apoptotic changes, initiation of endothelial active fatty acids metabolic reaction is thought to be an efficient strategy to reduce diabetic micro- and macro-vascular pathologies.^31^ However, cautions must be considered for the administration of Ex-4 in diabetic candidates. For example, prolonged and continuous subcutaneous use of EX-4 possibly causes nausea, vomiting and a negligible increase in the heart rate. In addition, systemic intravenous injection of this compound was found to yield severe tachycardia and arrhythmias in non-diabetic and diabetes cases in the swine model.^32^

## Effect of EX-4 on epitheliogenesis and cutaneous wounds

The accumulation of pro-oxidants and byproducts in squamous and non-squamous epithelial layers leads to cutaneous and gastric wounds in diabetic subjects.^33,34^ Lower-extremity amputation may be the only choice by the progression of diabetic foot ulcers after the onset of peripheral neuropathy, peripheral arterial disease, and immunosuppression.^34,35^ Epithelialization and wound repair provoke the recruitment of immune cells, fibroblasts, and keratinocytes and endothelial lineage. The successful cutaneous regeneration needs reciprocal cell-to-cell crosstalk through juxtacrine and paracrine interactions. Multiple growth factors such as vasculature endothelial growth factor, transforming growth factor-β (TGF-β), IL-1 and -6, tumor necrosis factor-α (TNF-α)) and other cytokines participate actively in epithelium repair.^36^ Systemic and topical administration of Ex-4 was found to decrease superoxide anions content and serum level of IL-6. Ex-4 has also angiogenesis potential in diabetic subjects. For example, it was found that the CD34^+^/KDR^+^ positive endothelial progenitor cells in the rat model of diabetes. Based on the data, the tubulogenesis rate increased in Ex-4-treated cells. In line with these finding, protein levels of VEGFR-2, matrix metalloproteinase-2 (MMP-2), p-eNOS, and TGF-β in these cells showing an increased angiogenesis rate. In addition to angiogenesis potential, keratinocyte proliferation rate was also induced in Ex-4 treated rats.^36^ In gastro-intestinal tract, the frequency of gastric ulcer is also diminished after administration of Ex-4. The suppression of inflammation rate and oxidative stress is prohibited in the periphery of ulcers. These features are found to confine the progression of ulcers and accelerate the healing.^33^

## Anti-cancer effect of Ex-4

T2DM has been shown to predispose the risk of many malignancies, such as colon and breast and other cancer types.^37,38^ Previous data demonstrated the anti-cancer properties of Ex-4 on cancer cell types such as breast cancer cells.^39^ As previously described, Ex-4 has the potential to inhibit breast cancer cell proliferation and metastasis and promote apoptosis by the modulation of effectors such as Caspase-9, Akt, and MMP-2. In addition, the clonogenic property of cancer cells by up-regulation of tissue inhibitor of metalloproteinases-1 and -2 expressions. It was elucidated that the exposure of tumor cells to Ex-4 has the potential to confine tumor mass via engaging GLP-1R signaling pathway. Moreover, Ex-4 has the potential to attenuate ovarian cancer cell proliferation with the induction of GLP-1R mRNA expression.^40^ The promotion of pro-inflammatory status governed by NF-κB seems to be a critical tumoricidal mechanism.^41^ In other conditions such as pancreas and prostate cancers, the size of tumors decreases after exposure to Ex-4. Ex-4 provokes CD8^+^ cytotoxic T cells and modulates the function of Foxp3^+^ regulatory T cells. These changes limit the expansion and progression of cancers.^42^ Based on data, the modulatory effect of Ex-4 is different regarding cancer type. For example, the cytotoxic effect of Ex-4 on prostate cancer is mediated by modulation of ERK-MAPK via the cAMP-PKA without affecting apoptotic signaling pathways. Therefore, cautions must be considered in cancer inhibition based on cancer phenotype.^43,44^ Insulin resistance is touted as one of the most metabolic disorders during the onset of cancers that lead to cachectic status.^45^ Previous studies noted that hyperinsulinemia in response to insulin resistance may lead to tumor expansion by increasing the hepatic IGF-1 level while protein content of insulin-like growth factor binding proteins 1 and 2 decreases.^46^ Irrespective of the reasons for induction of insulin production, high levels of insulin provokes estrogen bioactivity which in turn increases the risk of cancer formation in breast.^47^ It is suggested to apply a combined therapy for the better tumoricidal effect of Ex-4 in cancer candidates. Simultaneous application of metformin and Ex-4 was found to yield better therapeutic effects.^48^ Interestingly; Ex-4 sensitizes cancer cells to ionizing radiation through adenosine monophosphate activated protein kinase (AMPK) activation, contributing to the reduction of colony number and density via reduction of phosphorylated mTOR.^49^ The application of Ex-4 in endometrial cancer Ishikawa xenografts in nude mice yielded promising outcomes.^50,51^ Honors and Kinzig found the therapeutic effects of Ex-4 in preventing cancer-associated cachexia in male rats with Yoshida sarcoma.^52^ However, it seems that the Ex-4 modulatory effect was more prominent in cases with small-sized tumor masses compared to subjects with large sized cancers. Administration of Ex-4 is able to return insulin to basal levels in tumor-bearing candidates. These data stand for a fact that Ex-4 could be used as anti-cancer agent in diabetic subjects susceptible to incidence of different cancer types.

## Ex-4 on neurodegenerative disorders

The existence of receptor GLP-1R has been previously described in CNS.^53^ In addition to neuroprotective potential of Ex-4 on CNS by the inhibition of apoptosis, this factor is also able to pass across the blood-brain barrier and binds to GLP-1R followed by the activation of adenylyl cyclase, PKC and mitogen-activated protein signaling pathways could regulate brain bioactivity and memory function.^54^ The administration of Ex-4 was shown to promote SERCA expression through activation of PKA/cAMP signaling pathways and subsequently leads to inhibition apoptosis after the onset of spinal cord injury.^55^ The levels of pro-apoptotic effectors such as Bax, Caspase-3 and cytochrome C are decreased while the expression of Bcl-2.^56^ Therefore, it seems that the application of Ex-4 could reverse the detrimental effects of Ex-4 on acute CNS pathologies. Based on data, the onset of diabetic changes could predispose the possibility of degenerative diseases such as Alzheimer’s disease or Parkinson’s disease. Under the emergence of degenerative diseases, hyperinsulinemia and down-regulated insulin signaling *per se* are seen routinely in the context of CNS.^57^ The promotion of degenerative diseases contributes to the unusual aggregation of peptides and/or proteins in the specific regions of the brain. For instance, the deposition of Aβ forms plaques in the extraneuronal area as well as Tau deposited in the form of filaments inside neurons.^58^ The introduction of insulin causes to activation of PI3K/AKT and GSK-3β, as two main downstream players in the context of the brain. Ex-4 has the potential to decrease the phosphorylation of GSK-3β and thereby inhibits the bioactivity of tau kinase. Along with these changes, the aggregation of Tau protein is dramatically diminished followed by extracellular protein accumulation removal.^59^ Most notably, the administration of Ex-4 could directly increase insulin signaling pathways via phosphorylation of insulin receptor substrate 1 (IRS-1).^60^ The phosphorylation of IRS-1 phosphorylation *per se* increased phosphorylated levels of AKT and GSK-3β contributing to the dephosphorylation of protein tau.^61^ The application of Ex-4 in experimentally induced Parkinson’s disease by using 1-methyl-4- phenyl-1, 2, 3, 6-tetrahydropyridine in the model of a mouse, decreased the degeneration of dopaminergic neurons and secretion of MMP-3 released from microglia.^62^ The occurrence of ischemic changes and stroke has been reported in diabetic candidates after the endothelial cells’ injury. It seems that the application of Ex-4 in diabetic changes could inhibit endothelial injury by engaging the PI3K/p-Akt/Bcl-xl/Bcl-2 axis.^63^

## Modulatory effect of Ex-4 cardiovascular disease

It is mighty that diabetic patients are susceptible to cardiovascular disorders.^44^ Hyperglycemia has detrimental effects on cardiac structure and function thereby contributing to pathological cardiomyopathy, cardiac hypertrophy,^64^ interstitial fibrosis,^65^ as well as increased apoptosis and oxidative stress also reported after the onset of insulin resistance.^66^ The participation of distinct signaling pathways was reported in diabetic hearts. For instance, it has recently documented that the modulation of AMPK in the pathophysiology of cardiomyocytes metabolism.^67^ Therefore, pharmacological activation/inhibition of effector AMPK brings inevitable impacts on the status and intensity of cardiac injury exposed to various metabolic situations. In downstream pathways, AMPK affects the activity of the mTOR signaling pathway that acts as a cross-talk linker between different cell bioactivities.^68^ Similar to some mTOR blockers such as rapamycin, activation of GLP-1/GLP-1R axis phosphorylates AMPK and reduces hypertrophic stress.^69^ During the occurrence of myocardial infarction (MI), the reduction of Akt-1 and MAPK kinase-3 phosphorylation is seen in the infarcted zones. Ex-4 treatment was found to increase the phosphorylation of these factors and reduce the extent of aberrant remodeling and the number of apoptotic cells juxtaposed to infarct regions.^70^ Furthermore, Ex-4 has the potential to promote angiogenesis and neovascularization by the proliferation of vascular endothelial cells and α-smooth muscle actin positive cells toward injured cardiac tissue.^70^ In addition, it was shown that Ex-4 exerts a prophylactic role against myocardial injury via the activation of the Sirt1/PGC1α signaling pathway. The activation of this axis could promote mitochondrial function coincided with the suppression of oxidative stress insults.^71^ APPL1 plays versatile roles to inhibit cardiomyocytes apoptosis via the interaction with systemic adipokines, adiponectin. This interaction *per se* contributes to the promotion of AMPK and proliferator-activated receptor-α (PPAR-α). Under the occurrence if MI, Ex-4 decreases cardiac tissue apoptosis by amplifying the systemic adiponectin concentration and APPL1 activation and phosphorylation of AMPK.^72^ Other cardiovascular abnormalities mainly heart failure happens due to the disruption in calcium recycling. Ex-4 ameliorates Ca^2+^ homeostasis in HF subjects by promoting eNOS/cGMP/PKG axis and of restoration of SERCA2a activity. These changes reduce cytoplasmic Ca^2+^ content and activity of CaMKII.^24^

The uncontrolled proliferation and migration of vascular smooth muscle cells (VSMCs) are touted as one of the risk factors resulting in atherosclerosis. During the development of atherosclerotic plaques, VSMCs undergo phenotype shifting with an enhanced proliferation rate.^73^ Along with these changes, the dynamic of multiple factors mainly angiotensins are changed. Of note, angiotensin-II is the main peptide that part take in the progression of atherosclerotic pathologies by the induction of VSMCs proliferation and migration via controlling the enzymes catalyze the phosphorylation of extracellular signal-regulated kinase 1/2 and JNK.^74^ Scientific literature added a notion that Ex-4 administration inhibits Ang II-induced phosphorylation of ERK1/2 and JNK, improving hypertension and atherosclerosis in the candidate population.^75^ In addition to critical role of ERK1/2 and JNK in the dynamic growth of VSMCs, some studies have revealed that nuclear receptor superfamily like neuron derived orphan receptor 1 (NOR1), one of the key regulators of VSMCs proliferation during the occurrence of atherosclerosis, activity removal may lead to the control of neo-intima formation in injured vascular context. The incubation of VSMCs with Ex-4 precisely regulates the proliferation of these cells through inhibiting NOR1 promoter activity.^76^ In patients with pulmonary arterial hypertension (PAH), a serious disorder which affects near to 30% of patients suffering from congenital heart disease is originated from extreme pulmonary blood flow due to excessive drug use, a genetic mutation in different factors like bone morphogenetic protein receptor type II.^77,78^ By applying Ex-4, the improvement of PAH is initiated via the reduction of pro-inflammatory cytokines interleukin-1α and 1β in PAH rats. After incubation of PAH rats with Ex-4, the function of right ventricular function was restored the number of the amount of Smooth muscle myosin heavy chain class II and α-SMA.^79^

## Dynamic of Ex-4 in renal tissue

Diabetic nephropathy is the consequent of T2DM. The elevation of excessive glucose contents injures the renal filtration system, thereby contributing to the occurrence of proteinuria or macroalbuminuria.^80^ The promotion of diabetic nephropathy causes to chronic kidney disease by time, leading to renal insufficiency. The possibility of infectious nephritis is also reported in diabetic conditions.^81^ Commensurate with these data, lowering systemic glucose contents seems a priority in the control of nephropathy. The application of Ex-4 in diabetic nephropathy has shown satisfactory results rat models by the increase of fasting insulin levels.^82^ The serum levels of creatinine and blood urea nitrogen were also reduced after EX-4 administration. It seems that Ex-4 has the ability to alleviate oxidative stress via decreasing malondialdehyde production and decrease of lipid peroxidation rate while the bioactivity of superoxide dismutase and glutathione peroxidase were also induced.^83^ One of the most therapeutic aspects related to Ex-4 therapeutic effects correlates with the anti-inflammatory property via the control of TNF-α, IL-6, hypersensitive C-reactive protein and CCL5 as commonly described in end-stage renal failure.^84,85^ Scientific literature highlighted the potent role of Ex-4 in alleviating renal proximal tubular cell injury in diabetic mice. It was established that the levels of IL-1β, TNF-α and ROS content and malondialdehyde (MDA) levels were increased in renal tissue with the progression of disease. The development of diabetic condition predisposes the initiation of tubulointerstitial fibrosis that hastens renal failure.^86^ During T2DM hyperglycemic condition, the accumulation of extracellular matrix is extensively promoted in spaces between renal tubular epithelial cells. The occupation of connective tissue with the extracellular matrix can make multiple resident renal cells to release extracellular vesicles containing proteins, mRNA, and miRNA, contributing to the progression of fibrotic changes.^87^ The application of Ex-4 decreases diabetic-associated fibrosis by decreasing TGF-β. Along with these changes, the proliferation of fibroblasts and collagen deposition are decreased in renal niche.^85^ Another modulatory effect of Ex-4 in reducing diabetes associated fibrosis could be related to changes in extracellular vesicle cargoes. It seems that the type and intensity of specific miRNAs and factors are modulated that subsequently decreases diabetic-fibrosis.^88^

In addition to diabetes-related nephropathies, cardio-renal syndrome (CRS) is defined as a common disorder between heart and kidneys in which any abnormalities in one of these organs may impact other tissue and *vice versa*. CRS is classified into five subtypes (Table 2).^89^ As above-mentioned, T2DM is a major risk factor that affects both organs via engaging different mechanisms such as hypertension, oxidative stress or insulin resistance. For instance, T2DM-derived heart failure or left cardiac ventricular dysfunction causes renal abnormalities.^90^ Studies have confirmed that combined therapy of Ex-4 and melatonin can be effective in preserving renal and cardiac functionality in CRS subjected. Using Ex-4 and melatonin in a rat model of CRS, significant favor effects were observed in renal function via decreasing pro-inflammatory factors (TNF-α, NF-κB, MMP-9, RANTES, and iNOS) and oxidative stress (NOX-1, -2 and -4) markers. Besides, the synthesis of apoptosis (Bax, cleaved Caspase 3 and cleaved PARP), fibrosis (Samd3 and TGF-β) markers and DNA damage (γ-H2AX) is inhibited after Ex-4 and melatonin treatment. Pathological features were decreased after using Ex-4 and melatonin in CRS patients which indicated the effective impact of this method on kidney function.^91^

**Table 2:** Cardiorenal syndrome subtypes and explanations

**CRS type**	**Definition**
Type 1: Acute cardio-renal	Acute heart dysfunction leads to kidney injury or dysfunction
Type 2: Chronic cardio-renal	Chronic heart dysfunction leads to kidney injury or dysfunction
Type 3: Acute reno-cardiac	Acute kidney dysfunction leads to heart injury or dysfunction
Type 4: Chronic reno-cardiac	Chronic kidney dysfunction leads to heart injury or dysfunction
Type 5: Secondary CRS	Systemic conditions leading to simultaneous injury and/or dysfunction of heart and kidney

Furthermore, Ex-4 along with melatonin improved mitochondrial function and suppressed βMHC mRNA/protein expression, resulting in the alleviation of cardiac hypertrophy, is decreased. These findings indicated the compensatory role of Ex-4 and melatonin treatment on cardiac function.^92^

### Effect of Ex-4 in the function of hepatic tissue

Due to the high-rate metabolic activity of hepatic tissue, this tissue is eligible for different metabolic disorders and diseases. Non-alcoholic fatty liver diseases (NAFLDs) are clinically determined with the accumulation of byproduct three acyl glycerol (TAG) in the hepatic tissue that coincided with the development of intracytoplasmic lipid droplets affecting over 5% of total hepatocytes. Beside, an excessive lipid accumulation causes to hepatic cells lipotoxicity and oxidative stress via increased lipid peroxidation, mitochondrial insufficiency, and high content of ROS production.^93^ A close relation between T2DM and NAFLD has been previously well-established in which insulin-resistance and compensatory hyperinsulinemia are commonly seen by defective lipid metabolism and accumulation of triglyceride in hepatic tissue in patients with NAFLD or T2DM associate β-cell dysfunction.^94^

Along with these changes, NAFLD can lead to hepatic steatosis, NASH, hepatic cirrhosis, and hepatocellular carcinoma.^95^ In patients with T2DM, both glucose and lipid metabolisms are deteriorated, leading to the increase of plasma levels of low-density lipoprotein, and adversely reduction of high-density lipoprotein.^96^ In the case of NAFLD, the content of different serum transaminases is slightly raised with NAFLD and the administration of GLP-1R could decrease systemic transaminase levels via the promotion of hepatic lipid metabolism. One possible mechanism is that hepatic lipid metabolism is activated via triggering GLP-1R signaling cascade and the promotion of PPAR-α. These changes diminish the synthesis of apolipoprotein C which accelerates the degradation of fatty acid in plasma, after the use of Ex-4 and stimulation of GLP-1R signaling pathway.^97^ Experiments have shown that incubation of *ob/ob* mice with Ex-4 exerted anti-hepatic steatosis and -fibrosis capacities.^98^ The modulatory effect of Ex-4 on hepatic steatosis is done by reducing lipid droplets. The deposition of collagens along the hepatic sinusoids in the fibrotic liver is reduced after Ex-4 treatment. It was found that Ex-4 increased the reduced PPAR-α and glucose transporter 4 expressions in *ob/ob* mice.^98^ The activity of *FTO* correlates with energy homeostasis and the control of energy consumption. Tha is gene is commonly upregulated in NFLD candidates, contributing to oxidative stress indicated by an increased MDA production and decrease of superoxide dismutase activity. Meanwhile, the rate of lipogenesis is increased inside hepatocytes. Under these conditions, the basal dynamic of effectors such as PI3K and Akt is prohibited.

In NFLD animal models, the use of Ex-4 leads to alleviation of NFLD related complications via suppression of *FTO* expression and MDA production and restoration Superoxide dismutase activity.^99^ In addition, the expression of genes participating in fatty acid oxidation promotes hepatocyte functional behavior after phosphorylation of Akt.^99^ As above-mentioned, steatosis is routinely reported in the NFLD patients correlated with an increased lipogenesis rate and TAGs accumulation inside hepatocytes.^100^

It is thought that the promotion of Wnt/β-catenin signaling could prohibit adipogenesis by suppressing CCAAT/enhancer-binding proteins-induced PPARγ expression in which a decrease in the expression of β-catenin was unveiled in* in vitro* model of steatosis. Ex-4 has the ability to activate β-catenin which in turn stops lipogenesis by suppression of PPARγ and SREBP-1.^101^ The use of Ex-4 in steatoric patients remove excessive fatty acids via regulating lipid metabolism and reducing VLDL synthesis after suppression of genes such as peroxisome *Pgc1β*, transcription factor *Srebp-1c* and *Fasn*, *Dgat1*, and Apob. Fasn participates in *de novo* lipogenesis and *Dgat1* promotes the last steps in hepatic three glyceride synthesis.^102^

It was found that Sirt-1 expression is decreased in high-fat-diet animal models and Ex-4 could reverse the Sirt-1 function either in *in vivo* and *in vitro* modelscoincided with nicotinamide phosphoribosyl transferase (Nampt) activity. The Nampt/visfatin enzyme could catalyze the conversion of NAM toward NAD^+^ and is required for Sirt-1 activity.^103^

As previously mentioned, endoplasmic reticulum (ER) plays a critical role in lipid and protein biosynthesis.^104^ Disruption of ER homeostasis contributes to the accumulation of misfolded/unfolded proteins seen in obesity. Dysfunction of insulin function and T2DM also has a pivotal role in fatty liver disease development.^74^ Progressive ER stress contributes to the development of NFLD and nonalcoholic steatohepatitis by different molecular mechanisms, including lipogenesis, inflammation, apoptosis, and autophagy.^105^ Documents showed that Ex-4 can alleviate hepatic steatosis and ER stress via controlling lipin-1β/α ratio and lipid hemostasis through Sirt1 and AMPK signaling pathways.^106^ Hepatic glycoproteins like SEPP1 and fetuin-A are known as novel biomarkers who participated in insulin resistance and NFLD. Overactivity of these glycoproteins in palmitic acid-treated hepatocytes contributes to ER stress after Ex-4 treatment via activating Sirt-1 and AMPK expression.^107^

## Conclusion

According to versatile functions of Ex-4 in different tissues and similar activities to GLP-1, it seems that the use of Ex-4 is possibly helpful in the modulation of pathologies correlated with insulin dysfunction and the promotion of GLP-1R signaling pathway.

## Ethical Issue

Not applicable

## Conflict of Interest

The authors have no conflict of interest to declare.
